# 5-Methoxy­methyl-4-phen­oxy-1*H*-pyrazol-3-ol

**DOI:** 10.1107/S1600536809050302

**Published:** 2009-11-28

**Authors:** Tara Shahani, Hoong-Kun Fun, R. Venkat Ragavan, V. Vijayakumar, S. Sarveswari

**Affiliations:** a X-ray Crystallography Unit, School of Physics, Universiti Sains Malaysia, 11800 USM, Penang, Malaysia; b Organic Chemistry Division, School of Advanced Sciences, VIT University, Vellore 632 014, India

## Abstract

In the title compound, C_11_H_12_N_2_O_3_, the pyrazole ring system is essentially planar [maximum deviation = 0.002 (2) Å] and forms a dihedral angle of 66.93 (9)° with the benzene ring. In the crystal packing, pairs of inter­molecular N—H⋯O and O—H⋯N hydrogen bonds connect neighbouring mol­ecules into dimers, generating *R*_2_^2^(10) and *R*_2_^2^(8) ring motifs, respectively. The crystal structure is further stabilized by C—H⋯π inter­actions.

## Related literature

For the biological activity of pyrazoles, see: Genin *et al.* (2000 ▶); Hsu *et al.* (1956 ▶); Jung *et al.* (2002 ▶); Kudo *et al.* (1999 ▶); Singh *et al.* (1978 ▶); Skipper *et al.* (1955 ▶); Storer *et al.* (1999 ▶); Tewari & Mishra (2001 ▶). For pyrazole derivatives, see: Baraldi *et al.* (2003 ▶); Brown *et al.* (2004 ▶); Duma *et al.* (2000 ▶); Heerding (2003 ▶); Qiao *et al.* (2003 ▶); Stamford & Wu (2004 ▶). For a related structure, see: Goh *et al.* (2009 ▶). For hydrogen-bond motifs, see: Bernstein *et al.* (1995 ▶). For bond-length data, see: Allen *et al.* (1987 ▶). For the stability of the temperature controller used for the data collection, see: Cosier & Glazer (1986 ▶).
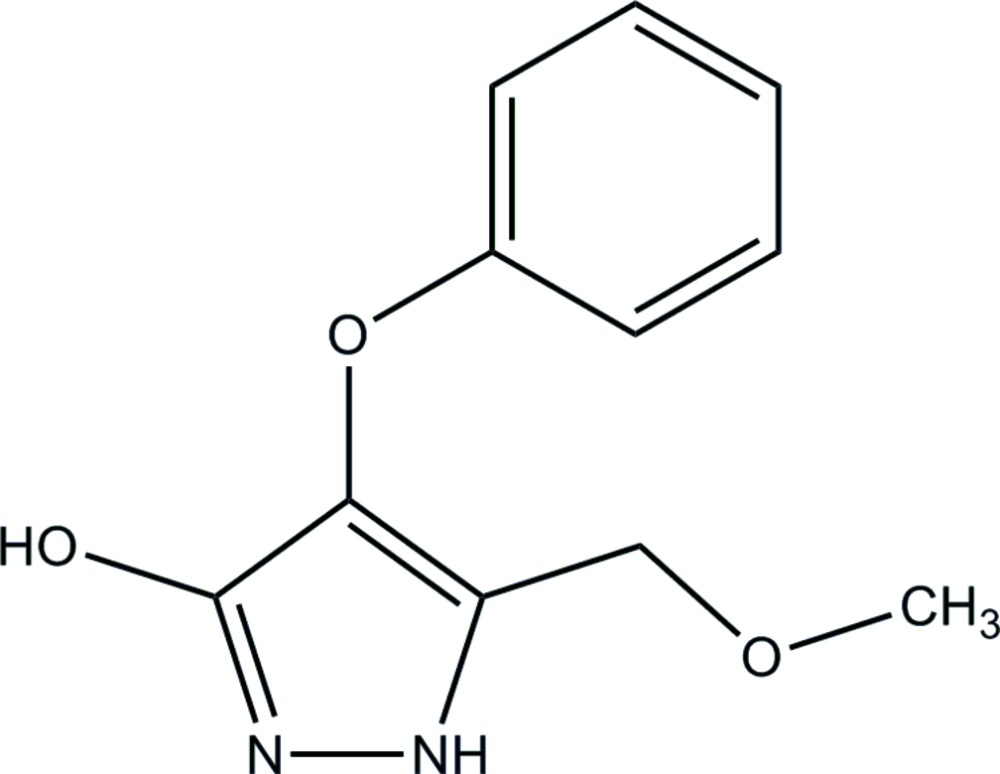


## Experimental

### 

#### Crystal data

C_11_H_12_N_2_O_3_
*M* *_r_* = 220.23
Monoclinic, 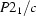

*a* = 8.8876 (5) Å
*b* = 10.3031 (5) Å
*c* = 12.0083 (6) Å
β = 100.917 (3)°
*V* = 1079.7 (1) Å^3^
*Z* = 4
Mo *K*α radiation
μ = 0.10 mm^−1^
*T* = 100 K
0.69 × 0.57 × 0.18 mm

#### Data collection

Bruker SMART APEXII CCD area-detector diffractometer
Absorption correction: multi-scan (**SADABS**; Bruker, 2005 ▶) *T*_min_ = 0.934, *T*_max_ = 0.983
14470 measured reflections
3433 independent reflections
2373 reflections with *I* > 2σ(*I*)
*R*_int_ = 0.032

#### Refinement

*R*[*F* ^2^ > 2σ(*F* ^2^)] = 0.058
*wR*(*F* ^2^) = 0.138
*S* = 1.10
3433 reflections
193 parameters
All H-atom parameters refined
Δρ_max_ = 0.45 e Å^−3^
Δρ_min_ = −0.33 e Å^−3^

### 

Data collection: *APEX2* (Bruker, 2005 ▶); cell refinement: *SAINT* (Bruker, 2005 ▶); data reduction: *SAINT*; program(s) used to solve structure: *SHELXTL* (Sheldrick, 2008 ▶); program(s) used to refine structure: *SHELXTL*; molecular graphics: *SHELXTL*; software used to prepare material for publication: *SHELXTL* and *PLATON* (Spek, 2009 ▶).

## Supplementary Material

Crystal structure: contains datablocks global, I. DOI: 10.1107/S1600536809050302/lh2960sup1.cif

Structure factors: contains datablocks I. DOI: 10.1107/S1600536809050302/lh2960Isup2.hkl

Additional supplementary materials:  crystallographic information; 3D view; checkCIF report

## Figures and Tables

**Table 1:** Hydrogen-bond geometry (Å, °)

*D*—H⋯*A*	*D*—H	H⋯*A*	*D*⋯*A*	*D*—H⋯*A*
N2—H1*N*2⋯O2^i^	0.91 (2)	1.89 (2)	2.7778 (18)	165.7 (19)
O3—H1*O*3⋯N1^ii^	0.92 (2)	1.74 (2)	2.6663 (18)	176 (2)
C3—H3*A*⋯*Cg*1^iii^	0.94 (2)	2.77 (3)	2.73	147.6 (18)

Symmetry codes: (i) 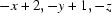; (ii) 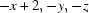; (iii) 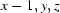. *Cg*1 is the centroid of the C1–C6 benzene ring.

**Fig. 1.**
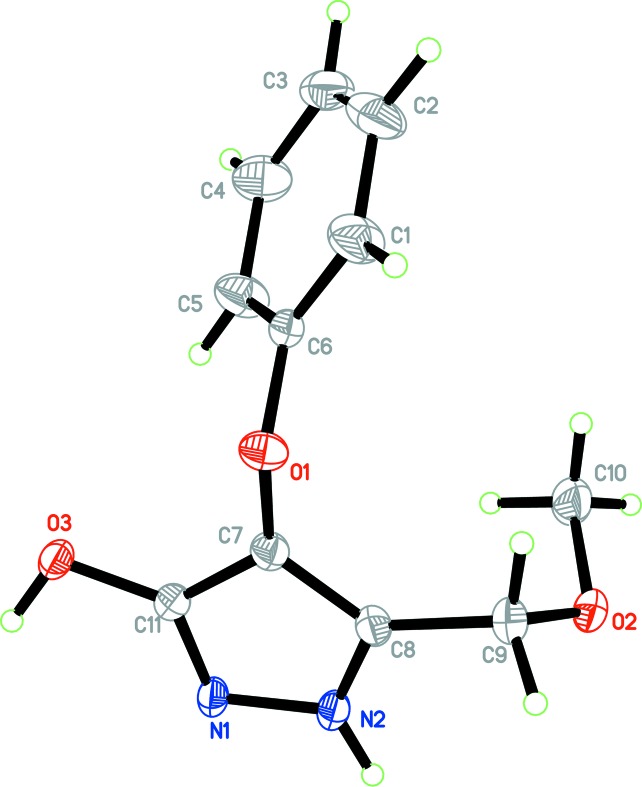
The molecular structure of the title compound, showing 50% probability displacement ellipsoids and the atom numbering scheme.

**Fig. 2.**
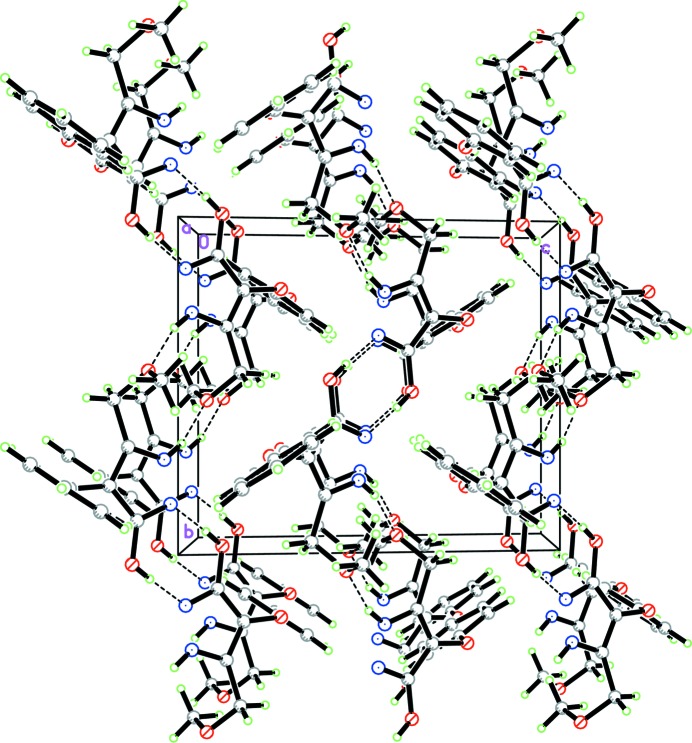
The crystal packing of the title compound, viewed along a axis. Intermolecular hydrogen bonds are shown by dashed lines.

## supplementary crystallographic information

### Comment

Pyrazoles are an important class of heterocyclic compounds and many pyrazole derivatives have a broad spectrum of biological activities such as anti-inflammatory (Singh *et al.*, 1978; Tewari & Mishra, 2001), anti-viral (Genin *et al.*, 2000; Storer *et al.*, 1999), anti-tumor (Hsu *et al.*, 1956; Skipper *et al.*, 1955), and herbicidal (Jung *et al.*, 2002; Kudo *et al.*, 1999) activities. Recently urea derivatives of pyrazole been reported as potent inhibitors of P^38^ kinase (Duma, 2000), On the other hand, pyrazole derivatives are anti-angiogenic agent (Qiao *et al.*, 2003), A3 adenosine receptor antagonist (Baraldi *et al.*, 2003), neuropeptide YY5 receptor antagonists (Stamford & Wu, 2004) and kinase inhibitor for the treatment of type 2 diabetes, hyperlipidemia and obesity (Brown *et al.*, 2004) as well as thrombopiotinmimetics (Heerding, 2003). Since the high electronegativity of halogens (particularly chlorine and fluorine) in the aromatic part of the drug molecules play an important role in enhancing their biological activity, we are interested to have 4-fluoro and 4-chloro substituted phenyl rings in the aromatic part of a 1,5-diaryl pyrazole. As part of our on going research programme aiming at the synthesis of new anti-microbial compounds, herein we report the crystal structure of a novel pyrazole derivative.

In the crystal structure (Fig. 1), the pyrazole ring system (C7/C8/N2/N1/C11) is approximately planar, with a maximum deviation of 0.002 (2) Å for atom C11. The dihedral angle formed between the mean plane of pyrazole ring and the benzene ring (C1–C6) is 66.93 (9)°. The bond lengths (Allen *et al.*, 1987) and angles are within normal ranges and comparable to a closely related structure (Goh *et al.*, 2009).

In the crystal packing (Fig. 2), pairs of intermolecular N2—H1N2···O2^i^ and O3—H1O3···N1^ii^ hydrogen bonds (Table 1) connect neighbouring molecules, into dimers, generating *R*_2_^2^(10) and *R*_2_^2^(8) ring motifs (Bernstein *et al.*, 1995), respectively. The crystal structure is further stabilized by C—H···π interactions (Table 1), involving the C1–C6 (centroid *Cg*1) benzene ring.

### Experimental

LiHMDS (19.4 ml, 1.0 min THF, 19.4 mmol) was added quickly to the solution of oxyacetic acid ethyl ester (1.0 g, 5.5 mmol) in toluene (15.0 ml) using syringe at 195 K with agitation and the anion formed was allowed to stand for approximately 1 min, and then 2-methoxyacetyl chloride (1.0 ml, 13.8 mmol) was added into the lot with stirring. Reaction mixture was removed from acetone-dry ice bath and stirred for 10 min then acetic acid (2.0 ml) was added with stirring. Ethanol (15.0 ml) and hydrazine hydrate (1.5 ml, 44.0 mmol) was added and refluxed for 10 min. Reaction mixture was concentrated to dryness under reduced pressure and redissolved in ethyl acetate. The organic layer was washed with saturated brine solution, dried over Na_2_SO_4_ and evaporated under reduced pressure. Crude product was purified by column chromatography using a mixture of 1:99 methanol and ethylacetate. Pale yellow solid was obtained. *Mp.* 418.8–419.8 K. Yield: 57%.

### Refinement

All hydrogen atoms were located in a difference map and were refined freely. [Range of C—H = 0.94 (2)–1.03 (2) Å].

### Crystal data

**Table:**

C_11_H_12_N_2_O_3_	*F*(000) = 464
*M**_r_* = 220.23	*D*_x_ = 1.355 Mg m^−^^3^
Monoclinic, *P*2_1_/*c*	Mo *K*α radiation, λ = 0.71073 Å
Hall symbol: -P 2ybc	Cell parameters from 6087 reflections
*a* = 8.8876 (5) Å	θ = 2.3–32.2°
*b* = 10.3031 (5) Å	µ = 0.10 mm^−^^1^
*c* = 12.0083 (6) Å	*T* = 100 K
β = 100.917 (3)°	Plate, yellow
*V* = 1079.7 (1) Å^3^	0.69 × 0.57 × 0.18 mm
*Z* = 4	

### Data collection

**Table:**

Bruker SMART APEXII CCD area-detector diffractometer	3433 independent reflections
Radiation source: fine-focus sealed tube	2373 reflections with *I* > 2σ(*I*)
graphite	*R*_int_ = 0.032
φ and ω scans	θ_max_ = 31.0°, θ_min_ = 2.3°
Absorption correction: multi-scan (*SADABS*; Bruker, 2005)	*h* = −12→11
*T*_min_ = 0.934, *T*_max_ = 0.983	*k* = −14→14
14470 measured reflections	*l* = −17→17

### Refinement

**Table:**

Refinement on *F*^2^	Primary atom site location: structure-invariant direct methods
Least-squares matrix: full	Secondary atom site location: difference Fourier map
*R*[*F*^2^ > 2σ(*F*^2^)] = 0.058	Hydrogen site location: inferred from neighbouring sites
*wR*(*F*^2^) = 0.138	All H-atom parameters refined
*S* = 1.10	*w* = 1/[σ^2^(*F*_o_^2^) + (0.0465*P*)^2^ + 0.7446*P*] where *P* = (*F*_o_^2^ + 2*F*_c_^2^)/3
3433 reflections	(Δ/σ)_max_ < 0.001
193 parameters	Δρ_max_ = 0.45 e Å^−^^3^
0 restraints	Δρ_min_ = −0.33 e Å^−^^3^

### Special details

**Table:**

Experimental. The crystal was placed in the cold stream of an Oxford Cyrosystems Cobra open-flow nitrogen cryostat (Cosier & Glazer, 1986) operating at 100.0 (1) K.
Geometry. All e.s.d.'s (except the e.s.d. in the dihedral angle between two l.s. planes) are estimated using the full covariance matrix. The cell e.s.d.'s are taken into account individually in the estimation of e.s.d.'s in distances, angles and torsion angles; correlations between e.s.d.'s in cell parameters are only used when they are defined by crystal symmetry. An approximate (isotropic) treatment of cell e.s.d.'s is used for estimating e.s.d.'s involving l.s. planes.
Refinement. Refinement of *F*^2^ against ALL reflections. The weighted *R*-factor *wR* and goodness of fit *S* are based on *F*^2^, conventional *R*-factors *R* are based on *F*, with *F* set to zero for negative *F*^2^. The threshold expression of *F*^2^ > σ(*F*^2^) is used only for calculating *R*-factors(gt) *etc*. and is not relevant to the choice of reflections for refinement. *R*-factors based on *F*^2^ are statistically about twice as large as those based on *F*, and *R*- factors based on ALL data will be even larger.

### Fractional atomic coordinates and isotropic or equivalent isotropic displacement parameters (Å^2^)

**Table:**

	*x*	*y*	*z*	*U*_iso_*/*U*_eq_	
O1	0.79382 (13)	0.20467 (12)	0.25817 (9)	0.0209 (3)	
O2	0.84461 (13)	0.54359 (11)	0.06550 (11)	0.0243 (3)	
O3	0.86330 (14)	−0.01790 (11)	0.10686 (10)	0.0210 (3)	
N1	0.97188 (15)	0.15166 (12)	0.01762 (11)	0.0187 (3)	
N2	0.98156 (16)	0.28291 (13)	0.03420 (12)	0.0197 (3)	
C1	0.5751 (2)	0.2793 (2)	0.32287 (16)	0.0313 (4)	
C2	0.4180 (2)	0.2854 (2)	0.31444 (19)	0.0373 (5)	
C3	0.3212 (2)	0.2243 (2)	0.22663 (17)	0.0315 (4)	
C4	0.3817 (2)	0.1569 (2)	0.14638 (17)	0.0339 (4)	
C5	0.5396 (2)	0.1508 (2)	0.15308 (16)	0.0293 (4)	
C6	0.63487 (18)	0.21173 (15)	0.24200 (13)	0.0182 (3)	
C7	0.85883 (17)	0.21114 (15)	0.16285 (13)	0.0178 (3)	
C8	0.91537 (18)	0.32155 (15)	0.12032 (13)	0.0188 (3)	
C9	0.9123 (2)	0.46065 (16)	0.15618 (14)	0.0222 (3)	
C10	0.6866 (2)	0.51602 (19)	0.02363 (19)	0.0310 (4)	
C11	0.89717 (17)	0.10779 (15)	0.09653 (13)	0.0177 (3)	
H1A	0.644 (3)	0.324 (2)	0.3821 (19)	0.042 (6)*	
H2A	0.375 (3)	0.334 (3)	0.368 (2)	0.056 (8)*	
H3A	0.214 (3)	0.227 (2)	0.2222 (19)	0.039 (6)*	
H4A	0.315 (3)	0.112 (3)	0.087 (2)	0.050 (7)*	
H5A	0.583 (2)	0.106 (2)	0.0976 (18)	0.033 (6)*	
H9A	1.017 (2)	0.4927 (19)	0.1791 (16)	0.021 (5)*	
H9B	0.853 (2)	0.466 (2)	0.2184 (17)	0.028 (5)*	
H10A	0.678 (2)	0.428 (2)	−0.0107 (19)	0.038 (6)*	
H10B	0.652 (3)	0.582 (3)	−0.032 (2)	0.047 (7)*	
H10C	0.625 (3)	0.518 (2)	0.088 (2)	0.041 (6)*	
H1N2	1.038 (2)	0.329 (2)	−0.0073 (17)	0.024 (5)*	
H1O3	0.924 (3)	−0.065 (2)	0.067 (2)	0.047 (7)*	

### Atomic displacement parameters (Å^2^)

**Table:**

	*U*^11^	*U*^22^	*U*^33^	*U*^12^	*U*^13^	*U*^23^
O1	0.0193 (5)	0.0261 (6)	0.0184 (5)	0.0006 (5)	0.0062 (4)	−0.0018 (4)
O2	0.0232 (6)	0.0139 (6)	0.0362 (7)	−0.0005 (4)	0.0070 (5)	0.0036 (5)
O3	0.0265 (6)	0.0133 (5)	0.0264 (6)	−0.0028 (5)	0.0134 (5)	−0.0010 (4)
N1	0.0228 (7)	0.0116 (6)	0.0232 (6)	0.0002 (5)	0.0081 (5)	0.0000 (5)
N2	0.0233 (7)	0.0131 (6)	0.0246 (7)	−0.0006 (5)	0.0091 (5)	0.0009 (5)
C1	0.0302 (9)	0.0340 (11)	0.0320 (9)	−0.0006 (8)	0.0114 (7)	−0.0121 (8)
C2	0.0321 (10)	0.0416 (12)	0.0431 (11)	0.0074 (9)	0.0194 (9)	−0.0070 (9)
C3	0.0215 (8)	0.0372 (11)	0.0378 (10)	0.0046 (8)	0.0110 (7)	0.0117 (8)
C4	0.0228 (9)	0.0446 (12)	0.0332 (10)	−0.0023 (8)	0.0027 (7)	−0.0019 (9)
C5	0.0236 (9)	0.0364 (11)	0.0286 (9)	0.0012 (7)	0.0065 (7)	−0.0094 (8)
C6	0.0198 (7)	0.0143 (7)	0.0220 (7)	0.0012 (6)	0.0080 (6)	0.0024 (6)
C7	0.0187 (7)	0.0175 (7)	0.0178 (7)	0.0007 (6)	0.0051 (5)	−0.0002 (6)
C8	0.0191 (7)	0.0154 (7)	0.0218 (7)	0.0013 (6)	0.0038 (6)	−0.0012 (6)
C9	0.0254 (8)	0.0153 (8)	0.0263 (8)	0.0004 (6)	0.0056 (6)	−0.0022 (6)
C10	0.0238 (9)	0.0213 (9)	0.0462 (11)	0.0011 (7)	0.0020 (8)	0.0031 (8)
C11	0.0181 (7)	0.0159 (7)	0.0198 (7)	−0.0007 (6)	0.0054 (5)	0.0001 (6)

### Geometric parameters (Å, °)

**Table:**

O1—C7	1.3781 (18)	C3—C4	1.377 (3)
O1—C6	1.3910 (19)	C3—H3A	0.94 (2)
O2—C9	1.425 (2)	C4—C5	1.392 (3)
O2—C10	1.427 (2)	C4—H4A	0.96 (3)
O3—C11	1.3406 (19)	C5—C6	1.382 (2)
O3—H1O3	0.92 (3)	C5—H5A	0.95 (2)
N1—C11	1.335 (2)	C7—C8	1.380 (2)
N1—N2	1.3672 (19)	C7—C11	1.410 (2)
N2—C8	1.343 (2)	C8—C9	1.498 (2)
N2—H1N2	0.91 (2)	C9—H9A	0.98 (2)
C1—C6	1.380 (2)	C9—H9B	0.99 (2)
C1—C2	1.383 (3)	C10—H10A	0.99 (2)
C1—H1A	0.96 (2)	C10—H10B	0.96 (3)
C2—C3	1.380 (3)	C10—H10C	1.03 (2)
C2—H2A	0.95 (3)		
			
C7—O1—C6	117.13 (12)	C1—C6—O1	116.36 (15)
C9—O2—C10	113.24 (13)	C5—C6—O1	122.80 (14)
C11—O3—H1O3	107.0 (15)	O1—C7—C8	125.92 (14)
C11—N1—N2	104.93 (13)	O1—C7—C11	128.09 (14)
C8—N2—N1	112.37 (13)	C8—C7—C11	105.64 (14)
C8—N2—H1N2	129.8 (13)	N2—C8—C7	106.50 (14)
N1—N2—H1N2	117.5 (13)	N2—C8—C9	122.63 (14)
C6—C1—C2	119.29 (18)	C7—C8—C9	130.87 (15)
C6—C1—H1A	119.0 (14)	O2—C9—C8	112.47 (13)
C2—C1—H1A	121.7 (14)	O2—C9—H9A	104.7 (11)
C3—C2—C1	120.65 (18)	C8—C9—H9A	109.7 (11)
C3—C2—H2A	119.0 (16)	O2—C9—H9B	109.6 (12)
C1—C2—H2A	120.3 (16)	C8—C9—H9B	107.9 (12)
C4—C3—C2	119.72 (17)	H9A—C9—H9B	112.5 (16)
C4—C3—H3A	119.8 (14)	O2—C10—H10A	108.6 (13)
C2—C3—H3A	120.5 (14)	O2—C10—H10B	105.4 (14)
C3—C4—C5	120.34 (18)	H10A—C10—H10B	111.7 (19)
C3—C4—H4A	119.9 (15)	O2—C10—H10C	111.1 (13)
C5—C4—H4A	119.7 (15)	H10A—C10—H10C	108.8 (18)
C6—C5—C4	119.20 (17)	H10B—C10—H10C	111.3 (19)
C6—C5—H5A	119.6 (13)	N1—C11—O3	122.92 (14)
C4—C5—H5A	121.2 (13)	N1—C11—C7	110.57 (14)
C1—C6—C5	120.79 (16)	O3—C11—C7	126.51 (14)
			
C11—N1—N2—C8	−0.09 (17)	N1—N2—C8—C9	179.33 (14)
C6—C1—C2—C3	0.3 (3)	O1—C7—C8—N2	173.96 (14)
C1—C2—C3—C4	−0.2 (3)	C11—C7—C8—N2	0.26 (17)
C2—C3—C4—C5	−0.4 (3)	O1—C7—C8—C9	−5.4 (3)
C3—C4—C5—C6	0.9 (3)	C11—C7—C8—C9	−179.12 (16)
C2—C1—C6—C5	0.2 (3)	C10—O2—C9—C8	64.02 (19)
C2—C1—C6—O1	−177.32 (17)	N2—C8—C9—O2	56.1 (2)
C4—C5—C6—C1	−0.7 (3)	C7—C8—C9—O2	−124.58 (18)
C4—C5—C6—O1	176.57 (17)	N2—N1—C11—O3	179.16 (14)
C7—O1—C6—C1	−142.80 (16)	N2—N1—C11—C7	0.26 (17)
C7—O1—C6—C5	39.8 (2)	O1—C7—C11—N1	−173.85 (14)
C6—O1—C7—C8	95.50 (18)	C8—C7—C11—N1	−0.33 (18)
C6—O1—C7—C11	−92.22 (19)	O1—C7—C11—O3	7.3 (3)
N1—N2—C8—C7	−0.12 (18)	C8—C7—C11—O3	−179.19 (15)

### Hydrogen-bond geometry (Å, °)

**Table:**

*D*—H···*A*	*D*—H	H···*A*	*D*···*A*	*D*—H···*A*
N2—H1N2···O2^i^	0.91 (2)	1.89 (2)	2.7778 (18)	165.7 (19)
O3—H1O3···N1^ii^	0.92 (2)	1.74 (2)	2.6663 (18)	176 (2)
C3—H3A···Cg1^iii^	0.94 (2)	2.77 (3)	2.73	147.6 (18)

Symmetry codes: (i) −*x*+2, −*y*+1, −*z*; (ii) −*x*+2, −*y*, −*z*; (iii) *x*−1, *y*, *z*.
